# Berries derived Polyphenols and Bone Health: A Systematic Review

**DOI:** 10.3390/nu17213440

**Published:** 2025-10-31

**Authors:** Simone Perna, Giorgia F. Ruina, Asmita Acharya, Giuseppe Mazzola, Mariangela Rondanelli, Patrizia Riso

**Affiliations:** 1 Department of Food, Environmental and Nutritional Sciences (DeFENS), Division of Human Nutrition, Università degli Studi di Milano, 20133 Milan, Italy; giorgiafrancesca.ruina@studenti.unimi.it (G.F.R.); asmita.acharya@unimi.it (A.A.); patrizia.riso@unimi.it (P.R.); 2 Department of Public Health, Experimental and Forensic Medicine, University of Pavia, 27100 Pavia, Italy; giuseppe.mazzola02@universitadipavia.it (G.M.); mariangela.rondanelli@unipv.it (M.R.)

**Keywords:** berry polyphenols, anthocyanins, bone mineral density (BMD), osteoporosis prevention, osteoblast and osteoclast activity, oxidative stress, bone remodeling

## Abstract

**Background**: Oxidative stress and inflammation contribute to osteoporosis. Berries provide polyphenols especially anthocyanins that may modulate bone remodeling. This review is the first to synthesize evidence specifically on berries and bone health, integrating human, animal, and in vitro data under the GRADE framework. **Methods**: We systematically searched PubMed, Embase, Web of Science, Scopus, and the Cochrane Library through 23 April 2025 for human, animal, and in vitro studies on berries or berry-derived compounds and bone outcomes. Risk of bias was assessed with RoB 2.0, ROBINS-I, SYRCLE, and an adapted ToxRTool; certainty of human evidence was appraised with GRADE. **Results**: Nineteen studies were included (5 human, 9 in vivo, 5 in vitro). Observational cohorts linked higher anthocyanin intake with greater BMD. Small randomized trials suggested modest benefits of blackcurrant and blueberry on whole-body BMD, bone turnover markers, and calcium retention, while results for biomarkers were mixed. Animal models generally showed attenuation of ovariectomy- or age-related bone loss, and in vitro experiments indicated inhibition of osteoclastogenesis with stimulation of osteoblast activity. By GRADE, certainty was low–moderate for BMD, low for biomarkers, and very low for fractures. **Conclusions**: Berry polyphenols may support skeletal health via antioxidant and anti-resorptive mechanisms, but current clinical evidence is limited by small samples, heterogeneity, and lack of fracture outcomes. Larger, longer, standardized RCTs with exposure profiling are needed before dietary recommendations can be made.

## 1. Introduction

Osteoporosis is a progressive skeletal disorder characterized by low bone mineral density (BMD) and microarchitectural deterioration, which increases the risk of fragility fractures. It predominantly affects postmenopausal women and older adults, representing a major public health concern with high morbidity, mortality, and economic burden worldwide [1,2]. Current pharmacological therapies, while effective, are often associated with long-term side effects and suboptimal adherence, highlighting the need for safe and sustainable nutritional strategies to support bone health [2].

Globally, osteoporosis affects an estimated 200 million women, and approximately one in three women and one in five men over the age of 50 will experience an osteoporotic fracture in their lifetime; hip, spine, and wrist fractures account for the bulk of the burden and contribute substantially to disability-adjusted life years and healthcare costs. With population aging, the absolute number of individuals at risk is projected to rise markedly over the next decades [3].

Nutrition is a key modifiable factor in the prevention of osteoporosis. Beyond the well-established roles of calcium, vitamin D, and protein, increasing attention has been directed toward bioactive compounds with antioxidant and anti-inflammatory activity. Among these, polyphenols derived from berries have attracted particular interest. Berries are rich in anthocyanins, flavonols, phenolic acids, and tannins, which can modulate bone remodeling by promoting osteoblastogenesis and inhibiting osteoclast activity [4,5]. Their ability to counteract oxidative stress and chronic low-grade inflammation is especially relevant given the central role of these processes in postmenopausal bone loss [3].

Preclinical studies indicate that berry-derived polyphenols improve trabecular bone architecture, increase bone strength, and regulate pathways such as RANK/RANKL/OPG and SIRT1-mediated signaling, thereby balancing bone formation and resorption [6,7]. In animal models, supplementation with berry extracts has been associated with enhanced bone mineral content and favorable modulation of bone turnover markers [8]. Clinical evidence, although limited and heterogeneous, suggests that higher intake of berries or anthocyanin-rich diets may contribute to the maintenance of BMD and reduction in fracture risk, particularly in postmenopausal women [3,9]. Furthermore, berries provide a natural dietary source of polyphenols that may be incorporated into daily nutrition without the limitations of pharmacological treatments, thus representing a potentially sustainable approach to lifelong skeletal health promotion. This line of research links nutritional science with preventive medicine and may offer integrative strategies for osteoporosis care.

While prior reviews have examined polyphenols broadly, none have focused exclusively on berries, a unique food-based source of anthocyanins with plausible anti-resorptive and osteogenic properties.

This review is the first to synthesize evidence specifically on berries and bone health, integrating human, animal, and in vitro data under the GRADE framework. Previous reviews addressed polyphenols broadly, but none focused exclusively on berries as dietary sources of anthocyanins. This focus highlights a unique, food-based preventive approach.

## 2. Materials and Methods

### 2.1. Study Design

**Figure 1:**
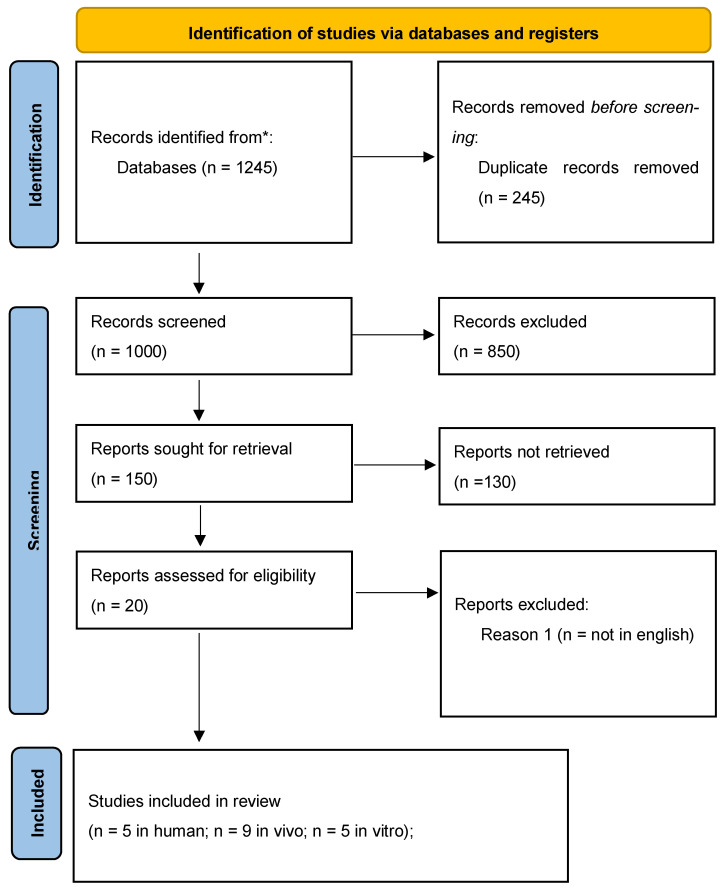
PRISMA 2020 flow diagram summarizing the study selection process for the systematic review on berry polyphenols and bone health.

This review followed PRISMA 2020. Although a meta-analysis was pre-specified, substantial heterogeneity in PICO (populations, berry species, doses, matrices, comparators, outcomes and DXA sites), measurement platforms, and follow-up precluded valid statistical pooling. We therefore performed a structured narrative synthesis with predefined subgroup considerations (e.g., intervention duration, dose range, DXA site). Screening and full-text review were conducted in duplicate with consensus/third-reviewer adjudication; the process is shown in Figure 1.

### 2.2. Eligibility Criteria

The studies that were included in this report are observational or interventional, focused on the impact of berries consumption on bone health in humans and non-human studies, and reported outcomes related to bone density or osteoporosis risk.

PICO/PECO (Population, Intervention/Exposure, Comparators, Outcome) criteria:

- Population: in human studies are included premenopausal women, twin pairs women and postmenopausal women (with or without osteoporosis/osteopenia…), in vivo studies involve OVX female rats to simulated age related bone loss, 20 days olds Sprague-Dawley rats to study early bone growth and young and old female C57BL/6J mice. In vitro studies include human bone marrow cells (pre-osteoclastic, CD34+, CD133), mouse bone marrow macrophages (BMMs), RAW264.7 cell line (a model for osteoclast studies), stromal cells from human exfoliated deciduous teeth (SHED) and MC3T3-E1 osteoblast.
- Intervention/Exposure: The studied exposures take into account the consumption of berries or the supplementation of specific polyphenols/berries extracts such as: Blueberry (blueberry extract, freeze dried blueberry powder, phenolic acids derived from blueberries), raspberry (whole fruit and specific fraction of rubus tozawae RL-Hex-NF3), blackcurrant (blackcurrant extract, powder and specific anthocyanins), blackberry (blackberries rich in cyanidin 3-O-β-D-glucoside and blackberries anthocyanins), cranberry (extract and A-type cranberry proanthocyanidins). The classes of polyphenols or specific compound involved in the studies are Flavonoids (catechin, procyanidin, flavanones, anthocyanins (such as pelargonidin, cyanidin, delphinidin, peonidin, petunidin, malvidin), Phenolic Acids (hydroxycinnamic acids (caffeic, ferulic), hydroxybenzoic acids (p-hydroxybenzoic, gallic, salicylic, vanillic, ellagic), Tannins (hydrolysable and ellagic tannins, condensed tannins), Petunidin.
- Comparators: used in these studies include placebo (for clinical trials), control groups (Sham, in animal models), no treatment (for clinical and animal studies), different doses, standard pharmacological treatments (for example estradiol and progesterone as a positive control in animal models) and baseline (measurements at the start of the study compared to the end).
- Outcomes: In this review, ‘relevant outcomes’ were pre-specified as: PRIMARY—areal BMD (DXA at lumbar spine, total hip, femoral neck, whole-body) and circulating bone turnover biomarkers (formation: P1NP, osteocalcin; resorption: CTX); SECONDARY—bone microarchitecture (e.g., BV/TV, Tb.N, Tb.Th), bone strength, calcium balance/retention, and molecular/cellular endpoints (e.g., RANKL/OPG, osteoclast/osteoblast assays). Human clinical outcomes were interpreted separately from animal and in vitro findings; no statistical pooling across models was attempted.

Studies were excluded if they were not published in English and if they were lacking relevant outcomes data. Data extraction was carried out independently by two reviewers using a tandardized form. Extracted information included study design, population characteristics, intervention or exposure details, comparators, and primary bone-related outcomes. Any discrepancies were resolved by discussion or by involvement of a third reviewer.

### 2.3. Search Strategy

Full, database-specific search strings (PubMed, Embase, Web of Science, Cochrane) and an a priori plan for grey literature (trial registries/Theses/ProQuest) have been applied. Google Scholar was used only for forward–backward citation chasing and not for primary study selection. English-language limits were applied due to resource constraints; this is acknowledged as a source of potential language bias.

The keywords that are used are related to berries, bone health, osteoporosis and dietary interventions, for example “bone density”, “osteoporosis prevention” and “dietary intervention on bone health”.

The search will use a combination of keywords and Medical Subject Headings (MeSH) terms to ensure comprehensive coverage of relevant literature. The following terms will be used:

*((“Berries”[Mesh] OR Vaccinium[tiab] OR Rubus[tiab] OR Fragaria[tiab] OR Ribes[tiab] OR Aronia[tiab] OR Sambucus[tiab] OR Euterpe[tiab] OR Aristotelia[tiab] OR Empetrum[tiab] OR Morus[tiab]*

OR berry*[tiab] OR blueberry*[tiab] OR bilberry*[tiab] OR blackberry*[tiab] OR raspberry*[tiab] OR strawberry*[tiab] OR cranberry*[tiab] OR lingonberry*[tiab] OR elderberry*[tiab]

OR açai[tiab] OR acai[tiab] OR maqui[tiab] OR chokeberry[tiab] OR currant*[tiab] OR gooseberry*[tiab] OR cloudberry*[tiab] OR boysenberry*[tiab] OR mulberry*[tiab]))

*AND*

*(“Bone Density”[Mesh] OR “Osteoporosis”[Mesh] OR “bone mineral density”[tiab] OR BMD[tiab] OR osteoporosis[tiab] OR osteopenia[tiab]*

*OR “bone turnover”[tiab] OR “C-terminal telopeptide”[tiab] OR CTX[tiab] OR P1NP[tiab] OR “Procollagen type I N-terminal propeptide”[tiab]*

*OR osteocalcin[tiab] OR RANKL[tiab] OR OPG[tiab] OR sclerostin[tiab]))*

### 2.4. Risk of Bias Assessment

The risk of bias of the included studies was independently assessed by two reviewers, with disagreements resolved by consensus or by consultation with a third reviewer. Different validated tools were applied according to the study design:

- Randomized controlled trials (RCTs): assessed using the revised Cochrane Risk of Bias tool for randomized trials (RoB 2.0) [10]. This tool evaluates domains such as the randomization process, deviations from intended interventions, missing outcome data, measurement of outcomes, and selection of the reported results.
- Non-randomized and observational studies: assessed using the Risk of Bias in Non-randomized Studies of Interventions (ROBINS-I) tool [11], which considers confounding, selection bias, classification of interventions, deviations from intended interventions, missing data, measurement of outcomes, and selective reporting.
- Animal studies: assessed with the SYRCLE Risk of Bias tool [12], specifically developed for preclinical studies, which adapts the Cochrane domains to the context of animal experiments, including allocation concealment, blinding, and incomplete outcome data.
- In vitro studies: since no standardized tool exists, we applied an adapted version of the ToxRTool (Toxicological data Reliability Assessment Tool), which evaluates the reliability of toxicological and mechanistic studies based on reporting quality, methodological adequacy, and plausibility of results [13]. Studies were classified as “reliable without restriction,” “reliable with restrictions,” or “not reliable.”

Risk of bias

**Figure 2:**
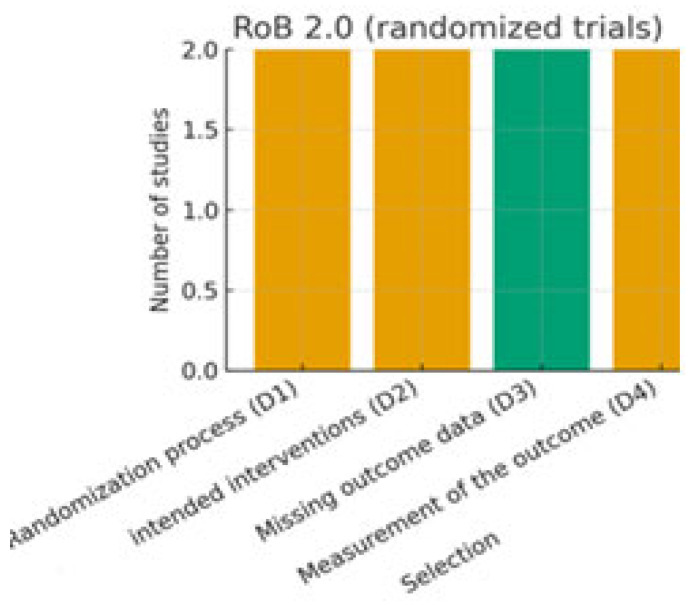
Risk of bias assessment for randomized controlled trials using the RoB 2.0 tool.

**Figure 3:**
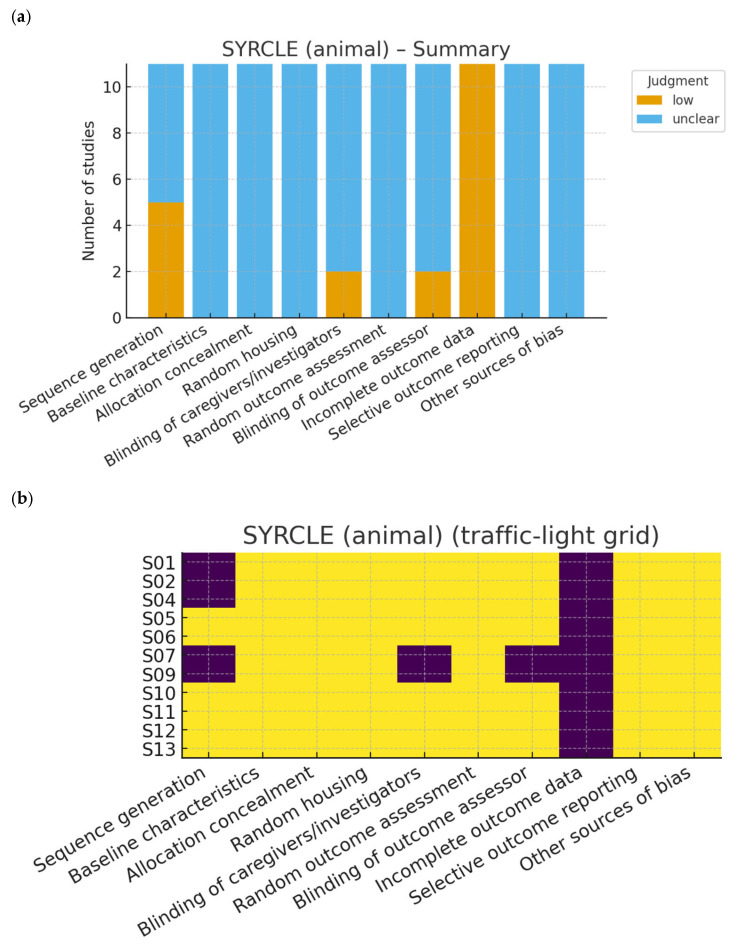
(**a**) Risk of bias assessment for animal studies using the SYRCLE tool and (**b**) Traffic-light plot of the SYRCLE risk of bias assessment for individual animal studies, illustrating the distribution of low, unclear, and high risk across domains.

**Figure 4:**
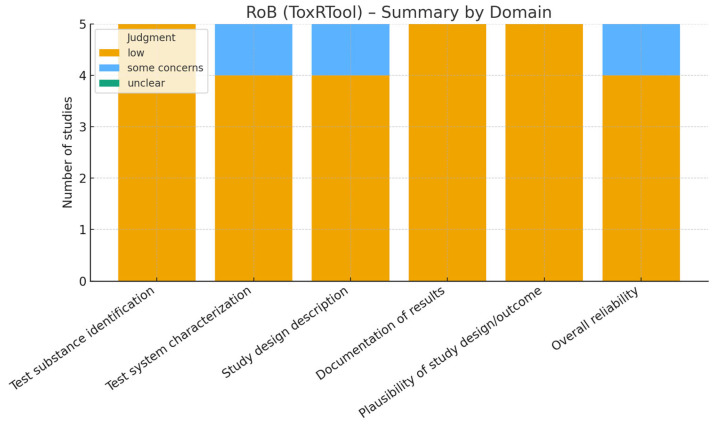
Risk of bias assessment for in vitro studies using the adapted ToxRTool.

Risk of bias was assessed independently by two reviewers after calibration, with disagreements resolved by a third reviewer. Domain-level decision rules were pre-specified and aligned with GRADE downgrading domains (risk of bias, inconsistency, indirectness, imprecision, publication bias). Detailed, study-level judgments are reported with traffic-light plots in Figure 2, Figure 3 and Figure 4. This approach allowed for a transparent evaluation of the internal validity and reliability of evidence across the different experimental models included in this systematic review. For SYRCLE assessments in animal studies, ‘unclear’ ratings most commonly reflected missing information on sequence generation, allocation concealment, random housing, blinding of caregivers/outcome assessors, and sample-size calculation. Where housing was described but randomization was not explicitly stated, we judged selection bias domains as ‘unclear’ rather than ‘low’. This underreporting limits internal validity and contributed to cautious interpretation.

## 3. Results

### 3.1. Characteristics of Included Studies

Finally, 19 studies met the inclusion criteria and were included in the systematic review, comprising 5 clinical studies, 9 preclinical animal studies, and 5 in vitro inves-tigations.

### 3.2. Human Studies

Evidence from human research on the effects of berry-derived polyphenols on bone health derives from both observational cohorts and clinical interventions. These two approaches provide complementary, though not always consistent, insights (Table 1).

Quantitative tabulation of key human studies (design, sample size, intervention, duration, and primary outcomes) is provided above to support interpretation.

Observational investigations, including large cohorts of women, consistently report that higher habitual intake of flavonoids, and particularly anthocyanins and procyanidins, is associated with higher BMD values at clinically relevant skeletal sites such as the femoral neck, lumbar spine, and hip [14,15]. These findings suggest that polyphenol-rich food sources such as berries may contribute to the preservation of bone mass over time. Nevertheless, observational designs are limited by their reliance on dietary self-reporting, residual confounding, and the absence of causal inference.

Interventional studies provide a more direct evaluation but are generally smaller in scale and shorter in duration. Nosal et al. (2022) [16] demonstrated that supplementation with blackcurrant powder for six months influenced BMD and modulated bone metabolism biomarkers in postmenopausal women, indicating a possible effect of anthocyanins on bone remodeling. Hodges et al. (2023) [18] showed that low to moderate doses of blueberry powder increased net bone calcium retention, supporting a potential role for berries in calcium balance and mineralization, although without consistent changes in circulating bone markers. In contrast, Hatcher (2017) [17] reported variable outcomes with raspberry supplementation, suggesting that not all berry species or compounds exert equivalent effects on bone turnover and density.

Overall, observational studies suggest long-term associations between berry-derived polyphenols and skeletal integrity, whereas clinical trials provide preliminary causal evidence but are limited by small cohorts, heterogeneous interventions, and short follow-up. The available evidence is supportive, but further well-designed randomized controlled trials are needed to clarify the magnitude and clinical relevance of these effects in osteoporosis prevention.

### 3.3. Animal Studies

Preclinical animal studies provide important mechanistic insights into the skeletal effects of berry-derived polyphenols and allow the evaluation of bone outcomes under controlled experimental conditions (Table 2). Most of the available work has been performed in ovariectomized rodent models, which mimic postmenopausal osteoporosis, although a few studies have focused on age-related bone loss or early-life interventions.

Blueberry supplementation has been the most frequently investigated approach. Studies in ovariectomized rats demonstrated that diets enriched with freeze-dried blueberry powder prevented whole-body BMD loss, improved bone mineral content, and reduced serum osteocalcin, suggesting a stabilization of bone turnover [21,25]. Additional work indicated that blueberry-derived phenolic acids promoted bone growth through activation of the p38 MAPK/β-catenin signaling pathway, enhancing osteoblast differentiation and impairing osteoclastogenesis [26]. Furthermore, early-life dietary exposure to blueberries was shown to prevent osteoblast senescence and reduce adult bone loss, emphasizing the potential long-term protective effects of berry polyphenols [27].

Other berry species have also been examined. Blackcurrant extracts attenuated trabecular and cortical bone loss in ovariectomized mice, improved trabecular bone volume, and enhanced antioxidant enzyme activity, thereby reducing oxidative stress–induced bone resorption [20,22]. Blackberry supplementation, particularly through cyanidin-3-O-β-D-glucoside, was found to improve bone mass and microarchitectural properties in rodents, further supporting the osteoprotective potential of anthocyanins [23]. More recently, fractions of *Rubus tozawae* (RL-Hex-NF3) demonstrated strong osteogenic effects by restoring bone density and enhancing markers of osteoblast differentiation in ovariectomized mice [24].

Collectively, animal studies indicate that berry supplementation helps preserve bone mass and architecture, primarily through antioxidant and osteogenic mechanisms. Anthocyanins and related compounds appear to modulate molecular pathways involved in bone remodeling. However, variability in dosage, intervention length, and berry preparations highlights the need for more standardized preclinical models to aid translation to human studies.

### 3.4. In Vitro Studies

In vitro studies have provided valuable mechanistic evidence regarding the cellular and molecular pathways through which berry-derived polyphenols influence bone metabolism (Table 3). These experiments typically employed osteoblast-like cells, osteoclast precursors, or mesenchymal stem cells, and tested purified berry compounds or polyphenol-rich extracts.

Cranberry extracts, particularly A-type proanthocyanidins, were shown to inhibit RANKL-dependent osteoclast differentiation, thereby reducing bone resorption activity in human bone marrow cells [28]. Consistent with this anti-resorptive activity, anthocyanin-rich fractions from blackcurrant and blackberry reduced osteoclast formation and activity while promoting osteoblast differentiation, indicating a dual role in shifting the balance toward bone formation [20]. Similarly, the anthocyanidin petunidin suppressed osteoclast differentiation in RAW264.7 cells by downregulating osteoclast-specific transcription factors and proteolytic enzymes [31].

On the osteogenic side, blueberry extracts were found to stimulate proliferation of human bone marrow progenitor cells while limiting osteoclastogenesis, suggesting a selective effect favoring anabolic processes [29]. Cranberry extracts were also reported to be biocompatible and to enhance osteogenic activity in stromal and osteoblast cell cultures, as evidenced by increased alkaline phosphatase activity, bone morphogenetic protein-2 expression, and matrix mineralization [30].

Overall, the in vitro results show that berry-derived polyphenols exert direct cellular effects that are consistent with the findings from animal and human studies. In vitro data suggest that berry compounds may influence oxidative stress, inflammatory signaling, and the regulation of osteoclast and osteoblast differentiation, thereby contributing to bone remodeling balance. However, the translation of these mechanistic insights to clinical practice requires careful consideration of bioavailability and effective concentrations achievable through diet.

### 3.5. Risk of Bias

The methodological quality of the included studies was assessed using tools appropriate for each study design. The PRISMA flow diagram summarizes the selection process (Figure 1), while graphical outputs illustrate the distribution of risk of bias across domains for randomized controlled trials, animal studies, and in vitro investigations (Figure 2, Figure 3 and Figure 4).

Among the human studies (Table 1), two randomized controlled trials were evaluated with the RoB 2.0 tool. As shown in Figure 2, both trials demonstrated a generally low risk of bias in the domains related to randomization, adherence to the intervention, and outcome measurement. Some concerns were identified for incomplete outcome data and selective reporting, which limited the overall certainty of the evidence. Observational cohorts were assessed with the ROBINS-I tool and were classified as having moderate risk of bias, mainly due to residual confounding and the inherent limitations of dietary self-reporting.

Animal studies (Table 2) were evaluated using the SYRCLE risk of bias tool. The majority presented an unclear risk of bias in domains such as allocation concealment, blinding of outcome assessment, and random housing (Figure 3a). Only a minority of studies provided sufficient details to be judged as low risk in domains such as sequence generation, blinding of caregivers, and random outcome assessment. None of the animal studies were rated as high risk, although the frequent lack of methodological reporting reduces confidence in reproducibility. The traffic-light grid (Figure 3b) provides an individual representation of the assessment, confirming that unclear reporting was the most common limitation across domains.

For in vitro studies (Table 3), an adapted version of the ToxRTool was applied. Most studies were considered “reliable with restrictions,” primarily due to incomplete reporting of details such as randomization of samples, blinding of assessors, and validation of exposure concentrations. Nevertheless, all were methodologically adequate to support mechanistic interpretations, particularly with regard to antioxidant and anti-resorptive effects of berry polyphenols.

In summary, the risk of bias assessment indicates that while the overall body of evidence is promising, the strength of conclusions is limited by small sample sizes, short durations, and incomplete reporting. The consistent direction of results across human, animal, and in vitro studies supports the biological plausibility of berries as osteoprotective agents, but future research requires more rigorous and standardized methodology.

**Figure 5:**
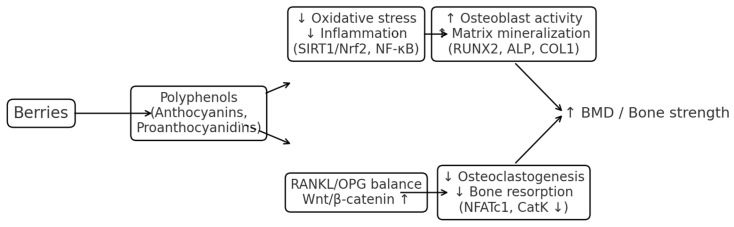
Proposed mechanism by which berry polyphenols may influence bone remodeling: dietary berries provide anthocyanins and related polyphenols that reduce oxidative stress and inflammatory signaling, modulate RANKL/OPG and Wnt/β-catenin pathways, suppress osteoclastogenesis, stimulate osteoblast activity, and ultimately support BMD and bone strength. ↑: support; ↓: suppress.

We hypothesize that inter-individual variability in response reflects differences in metabolism and gut microbiota processing of berry polyphenols. Stratified trials incorporating metabolomics and microbiome profiling should test the mechanistic pathway summarized in Figure 5 and evaluate whether responders can be prospectively identified.

### 3.6. GRADE Assestment for Human Studies

The certainty of evidence for human outcomes was assessed using the GRADE approach (Table 4). Risk-of-bias judgments were incorporated into GRADE as the primary reason for downgrading BMD and biomarker outcomes in humans.

Evidence linking higher anthocyanin intake with improved bone mineral density (BMD) was rated as low to moderate. The main reasons for downgrading were residual confounding in observational cohorts and small sample sizes with short follow-up periods in randomized controlled trials. The certainty of evidence for bone metabolism biomarkers was rated as low, due to heterogeneity of interventions and inconsistent results across studies. Evidence for fracture prevention was rated as very low, as no human trial directly assessed this outcome.

The GRADE framework was not applied to animal and in vitro studies, as these models are not formally covered by the system, which is designed for evaluating clinical and epidemiological evidence. Nonetheless, these studies provide mechanistic support that complements the human findings (Table 5).

## 4. Discussion

Compared with prior reviews on polyphenols and bone health, this work uniquely focuses on berries and applies GRADE to human evidence. The convergent findings across models strengthen biological plausibility, but methodological weaknesses (small n, short duration, incomplete reporting) lower certainty. Clinically, achievable berry intakes could provide supportive benefits, but they remain adjuncts to established osteoporosis prevention strategies (calcium, vitamin D, exercise). Future RCTs should use standardized berry preparations, assess bioavailability, and evaluate fracture outcomes.

In humans, observational cohorts consistently reported associations between higher dietary anthocyanin intake and greater bone mineral density at key skeletal sites [14,15]. These findings were supported by interventional data, in which blackcurrant powder supplementation improved bone metabolism biomarkers and immune-inflammatory status [16], and blueberry powder enhanced net calcium retention [18]. Comparable trends have been observed in other polyphenol-focused dietary studies, which collectively suggest a role for plant bioactives in bone remodeling [3,8]. Nevertheless, heterogeneity in study design, intervention duration, and dosage complicates the interpretation of clinical outcomes, and not all interventions demonstrated consistent benefits [17]. According to the GRADE assessment, certainty of evidence was rated as low to moderate for BMD, low for bone turnover biomarkers, and very low for fracture outcomes, highlighting the preliminary nature of these findings. Such variability is likely explained by differences in trial design, product standardization, and outcome assessment, and exploratory subgroup analyses did not resolve the observed inconsistency.

Preclinical models consistently supported the osteoprotective potential of berries. Evidence from animal experiments showed that blueberry supplementation may reduce bone loss and preserve trabecular architecture, although these effects were documented in short-term studies under controlled conditions and may not fully translate to humans [25,26,27]. Blackcurrant extracts attenuated trabecular bone loss and enhanced antioxidant enzyme activity, reinforcing the hypothesis that reduced oxidative stress may underlie skeletal benefits [20,22]. Pathways such as SIRT1 and Wnt/β-catenin are often implicated in polyphenol–bone interactions, but current evidence does not clarify whether the effects of berries are specific or reflect general polyphenol actions [6]. Furthermore, *Rubus tozawae* fractions promoted osteoblast differentiation and increased markers including alkaline phosphatase and type 1 collagen, suggesting a potential role for certain berry compounds as osteogenic modulators [24]. Most animal studies, however, presented unclear risk of bias across multiple SYRCLE domains, with few reporting sample-size calculations, randomization methods, allocation concealment, or blinded outcome assessment. This lack of methodological detail reduces confidence in reproducibility and contributed to downgrading for indirectness and risk of bias when translating findings to humans.

In vitro findings provided additional mechanistic support. Anthocyanin-rich extracts from cranberry, blackcurrant, and blueberry inhibited RANKL-mediated osteoclastogenesis while promoting osteoblast proliferation and differentiation [20,28,29]. The anthocyanidin petunidin further downregulated osteoclast-related gene expression, while cranberry extracts enhanced osteogenic markers in stromal and osteoblast cell lines [30,31]. These cellular findings align with recent reviews emphasizing the anti-resorptive and antioxidant properties of berry polyphenols in bone biology [4,5].

Despite encouraging signals, clinical applicability remains uncertain. Most human studies focused on surrogate endpoints such as BMD or turnover markers rather than fractures, and bioavailability of berry-derived compounds is variable across species, matrices, and preparations. Standardized interventions with characterized anthocyanin profiles, pharmacokinetic evaluation of parent compounds and metabolites, and longer follow-up are needed to determine whether surrogate changes translate into fracture risk reduction.

In conclusion, across human, preclinical, and in vitro evidence, berry-derived polyphenols appear biologically plausible contributors to skeletal health. Human studies provide preliminary associations, preclinical models elucidate potential mechanisms, and in vitro work offers molecular explanations. Yet, consistent with the GRADE evaluation, the overall certainty of evidence remains low to moderate for BMD, low for biomarkers, and very low for fracture outcomes. While the direction of findings is convergent, their robustness and clinical applicability are still uncertain.

### 4.1. Limitations

Several limitations should be considered when interpreting the findings of this review. First, most human studies were observational or small-scale trials, many of which relied on food frequency questionnaires, making them vulnerable to recall bias and measurement error. Dietary exposures in observational cohorts were primarily ascertained by food-frequency questionnaires, introducing measurement error and potential misclassification. Single baseline assessments increase susceptibility to regression dilution bias, as dietary patterns may change over time. Despite multivariable adjustment, residual confounding (e.g., overall diet quality, physical activity, health-seeking behaviors) cannot be excluded and may partly account for observed associations. The limited number of randomized controlled trials and their short duration prevent definitive conclusions on the long-term effects of berry consumption on fracture risk.

Second, translation from animal to human models remains a challenge, as differences in metabolism, gut microbiota interactions, and dosages of berry extracts may influence the extrapolation of results. Animal studies support antioxidant and anti-resorptive mechanisms, but the concentrations of anthocyanins used experimentally are often higher than those achievable through a normal diet.

Third, in vitro experiments, although useful for identifying specific molecular pathways, cannot fully replicate the complexity of bone physiology, where endocrine, immune, and nutritional factors interact simultaneously. The bioavailability and metabolism of polyphenols in humans further complicate the applicability of these findings.

Fourth, preclinical results should be interpreted cautiously given small sample sizes, limited reporting of randomization/blinding, and potential species-, sex-, and dose-specific effects. Dose translation from animals to humans and differences in metabolism and gut microbiota further limit direct extrapolation.

Finally, most of the included studies evaluated short- to medium-term outcomes. Longitudinal data on the sustained impact of berry consumption on BMD and fracture incidence are lacking, which remains a critical gap in the literature.

### 4.2. Future Implications

The findings of this review indicate that berry-derived polyphenols may have a potential role in nutritional strategies aimed at supporting bone health, although the current evidence is preliminary and heterogeneous. Future research should prioritize large, well-designed randomized controlled trials with standardized berry interventions, clear characterization of polyphenol content, and long-term follow-up to evaluate clinically relevant outcomes such as fracture incidence.

Further work is also required to clarify the dose–response relationship and the bioavailability of specific berry compounds. This may involve identifying the most relevant anthocyanins, assessing their metabolism in humans, and developing approaches that could improve their absorption and stability.

From a broader perspective, incorporating berries into the diet could be considered as one of several possible approaches to support skeletal health, but available data are insufficient to support firm recommendations. Evidence from animal studies suggesting benefits of early-life interventions requires careful validation in human trials. Clinically, berry intake should be regarded as complementary to established measures such as adequate calcium and vitamin D intake and regular physical activity, which remain the cornerstone of osteoporosis prevention.

Anthocyanins exhibit low oral bioavailability, and circulating exposure is dominated by phase II conjugates (glucuronides, sulfates) and gut-derived phenolic acids. Consequently, the micromolar concentrations used in vitro and the high per-kg doses in animal models exceed circulating levels achievable through habitual berry intake. This gap is a major translational bottleneck. Future RCTs should include pharmacokinetic profiling (parent compounds and key metabolites), assess food matrix effects, and relate exposure to bone endpoints.

To support translation, future trials should pre-define diet-achievable exposure ranges (e.g., 50–150 g/day whole berries or matched extract providing ~150–300 mg/day anthocyanins as cyanidin-3-glucoside equivalents), incorporate pharmacokinetic profiling of parent compounds and key metabolites, and relate exposure to BMD/biomarker responses.

## 5. Conclusions

Berry-derived polyphenols show potential to preserve bone mass through antioxidants and anti-resorptive pathways. Current human evidence remains preliminary, with low certainty for BMD and very low certainty for fractures. Until large, long-term RCTs are available, berries should be considered as complementary, rather than primary strategies for bone health.

## Figures and Tables

**Table 1:** Summary of human studies evaluating the effects of berry-derived polyphenols on bone mineral density, bone metabolism biomarkers, and osteoporosis-related outcomes.

Paper	Area of Interest	Compound	Sample	Dosage/Posology	Mechanism	Main Results
**Hardcastle et al., 2011** [14]	Bone Health (BMD) measurement at femoral neck and lumbar spine)	Flavonoids (Catechin, Procyanidin, Flavanones)	Premenopausal women (n = 2929)	Diets via FFQ analyzed for flavonoid intake	Catechin e procyanidin: associated with increased BMD; Flavanones: no effect	Catechin and procyanidin associated with increased BMD; Flavanones showed no effect
**Welch et al., 2012** [15]	Association between habitual flavonoid intake and BMD	Flavonoids (anthocyanins, flavanones)	Women (twins) (n = 3160)	Habitual intake assessed via FFQ	Not specified (observational)	Anthocyanins associated with the highest observed BMD; high flavanone intake positively associated with hip BMD
**Nosal et al.,** **2022** [16]	Postmenopausal osteoporosis	Blackcurrant powder	Randomized, double-blind, placebo-controlled clinical trial on postmenopausal women (n = 40 in the final analysis). Groups: placebo (Control, n = 13), 392 mg/day (low BC, n = 16), 784 mg/day (high BC, n = 11)	392 mg/day and 784 mg/day for 6 months	Not specified in detail	Changing in whole body bone mineral (BMD) from baseline after 6 months and effect of different doses of blackcurrant powder on bone metabolism biomarkers and immuno-inflammatory status.
**Hatcher, 2017** [17]	Bone health, osteoporosis, bone mineral density (BMD)	Raspberry	Postmenopausal women (placebo and raspberry groups)	Quantity not specified	Effects on bone formation and resorption biomarkers	Effects on bone mineral density of the radius, femoral, neck and total body baseline and at the end of the study
**Hodges et al., 2023** [18]	Bone calcium balance, net bone calcium retention	Freeze-dried blueberry (BB)	Clinical study with 14 postmenopausal women	Human study: low (17.5 g/day), medium (35 g/day), and high (70 g/day) of BB powder	Measurement of urinary 41Ca:Ca ratio in women to assess net bone calcium retention. Secondary assessment of bone metabolism biomarker	increase in net bone calcium retention with low and medium doses of BB powder compared to no treatment. No significant differences in serum calcium or 25(OH)D levels between interventions. Markers of bone resorption and osteocyte activity did not show significant changes with BB consumption

**Table 2:** Summary of preclinical animal studies investigating the effects of berry-derived polyphenols on bone mineral density, bone architecture, and markers of bone remodeling.

Paper	Area of Interest	Compound	Sample	Dosage/Posology	Mechanism	Main Results
**Li et al. (2014)** [19]	Prevention of osteoporosis induced by ovariectomy	Blueberry extract	Ovariectomized female Sprague Dawley rats	10% *w*/*w* freeze-dried blueberry powder in the diet	Inhibition of bone resorption	Blueberries inhibit bone resorption, bone loss, and reduction in bone strength in OVX rats.
**Zheng et al. (2016)** [20]	Reduction in trabecular and cortical bone loss	Blackcurrant extract	Ovariectomized female C57BL/6J mice	1% blackcurrant extract in the diet	Attenuation of bone resorption	Blackcurrant attenuates OVX-induced bone loss, as measured by BMD and trabecular volume; reduces bone resorption activity.
**Melough et al. (2017)** [21]	Impact of berries antioxidants on ovariectomy-induced bone loss	Blueberry	Ovariectomized Sprague Dawley female rats	5% *w*/*w* dried blueberry powder for 100 days	Prevention of BMD loss	Blueberry prevents OVX-induced loss of whole-body BMD; the blueberry-treated group has lower serum osteocalcin levels
**Sakaki et al. (2018)** [22]	Improvement of mice bone mass (age-related bone loss)	Blackcurrant extract	Young and old female C57BL/6J mice	1% (*w*/*w*) blackcurrant extract in chow diet for four months	Improved glutathione peroxidase and catalase activity, increased trabecular bone volume, OB surface, and bone mineral content	Consumption of blackcurrant early in life prevented ageing-associated bone loss in young mice; no effect in aged mice
**Kaume, L. K. (2012)** [23]	Bone mass and microarchitectural properties	Blackberries rich in cyanidin 3-O-β-D-glucoside	Ovariectomized (OVX) rats. Groups: Sham + Control (n = 12), OVX + Control (n = 11), OVX + BB 5% (5% *w*/*w* in the diet, n = 6), OVX + BB 10% (10% *w*/*w* in the diet, n = 7	5% and 10% (*w*/*w*) of blackberries in the die	Not specified in detail in the abstract/introduction	Blackberries consumption improved bone mass and microarchitectural properties in the OVX rat model
**Hong et al., 2024** [24]	Postmenopausal osteoporosis	Fraction of *Rubus tozawae* (RL-Hex-NF3)	8-week-old female C57BL/6J mice with ovariectomy (OVX). Groups: SHAM (n = 8), OVX (n = 8), E + P (0.1 mg/kg β-estradiol and 1 mg/kg progesterone, n = 8), RLL (10 mg/kg RL-Hex-NF3, n = 8), RLH (40 mg/kg RL-Hex-NF3, n = 8	10 mg/kg and 40 mg/kg of RL-Hex-NF3 administered orally for 12 weeks	Induction of osteoblast differentiation	Oral administration of RL-Hex-NF3 restored bone density in OVX mice. Increased serum levels of osteocalcin (OCN). Improved bone microarchitecture parameters (BMD, BV/TV, BS/BV, BS/TV, Tb.Th, Tb.N). Increased type 1 collagen (COL1) content in the femur and alkaline phosphatase (ALP) activity. Increased expression of osteoblast stimulators (β-catenin, TFG β, BMP2/4) and osteoblastic markers (OPN, OSX, RUNX2, COL1). Increased mRNA expression of *Alp*, *Runx2*, and *Ocn*. Three active compounds were identified in RL-Hex-NF3: 3β-hydroxy-18α,19α-urs-20-en-28-oic acid (1), betulinic acid (2), and (1S,6R,7S)-muurola-4,10(14)-dien-15-ol (3)
**Devareddy et al., 2008** [25]	Effect of blueberry on bone loss in ovariectomized rat model of postmenopausal osteoporosis	Blueberry	3-month-old female Sprague-Dawley rats with ovariectomy (Ovx). Groups: Sham (n = 10), Ovx (n = 10), Ovx + Blueberry (n = 6)	OVX + 5% blueberry for 100 days duration	Berries demonstrate a positive effect on bone metabolism due to their antioxidant power by phenolic content	OVX + 5% blueberry group increased whole body BMD and serum ALP
**Chen et al., 2010** [26]	Effect of blueberry on bone growth, BMD, BMC, osteoblast and osteoclast numbers.	Blueberry (phenolic acids derived from blueberries)	Sprague-Dawley male/female rats; 20 days old (n = 20)	Control vs. 10% blueberry for 40 days duration	Dietary-induced serum phenolic acids promote bone growth via the p38 MAPK/β-catenin canonical WNT signaling pathway, upregulating osteoblast differentiation and osteocalcin production; decreases RANKL mRNA expression and impairs osteoclastogenesis	Increases in bone mass, BMD, Bone Mineral Content (BMC). Associated with increases in osteoblast number and decreased osteoclast number
**Zhang et al., 2011** [27]	Effect of early life blueberry supplementation on preventing osteoblast senescence and adult bone loss	Blueberry diet	Sprague-Dawley female rats, 20 days old	10% blueberry diet fed rats only between postnatal day 20 and postnatal day 34	Preventing osteoblast senescence; anti-oxidative and anti-inflammatory properties of anthocyanins	Early blueberry supplementation prevented osteoblast senescence and adult bone loss. Increased trabecular bone volume, osteoblast number, bone formation rate, and osteocalcin levels

**Table 3:** Summary of in vitro studies evaluating the effects of berry-derived polyphenols on osteoblast and osteoclast differentiation, signaling pathways, and bone remodeling mechanisms.

Paper	Area of Interest	Compound	Sample	Dosage/Posology	Mechanism	Main Results
Tanabe et al., 2011 [28]	Effects of cranberry extract on bone degradation	Cranberry extract (A-type cranberry proanthocyanidins)	Human bone marrow cells (pre-osteoclastic)	10, 25, 50, 100 µg/mL for 4 days	Inhibits RANKL-dependent osteoclasts; impairs cell maturation and decreases bone resorption	Decreased rate of bone degradation by inhibiting RANKL-dependent osteoclasts.
Bickford et al., 2006 [29]	Effects of blueberry extract on human stem cell proliferation	Blueberry extract	Human bone marrow cells (CD34+ or CD133)	500 ng/mL for 72 h	Promotes proliferation of human bone marrow cells (osteoblast progenitor cells) and decreases osteoclastogenesis	Increased proliferation of human bone marrow cells, decreased TRAP staining and number of RANKL-dependent osteoclasts
Bauer et al., 2024 [30]	Osteogenesis	Cranberry extract	Stromal cells from human exfoliated deciduous teeth (SHED) and Osteoblasts MC3T3-E1 (in vitro)	Cranberry extract at 0.2 mg/mL, 2 mg/mL, 20 mg/mL, 50 mg/mL, 100 mg/mL, 150 mg/mL, 200 mg/mL, and 250 mg/m	Evaluation of biocompatibility and stimulation of osteogenic activity	Demonstration of biocompatibility and improving
Zheng et al. 2016 [20]	Osteoclastogenesis, osteoblast differentiation, ovariectomy-induced bone loss	Blackcurrant anthocyanins (BCA), blackberry anthocyanins (BKA), anthocyanin-rich blackcurrant extract	Mouse bone marrow macrophages (BMMs). In vivo: Ovariectomized female C57BL/6J mic	1 and 3 µg/mL of BCA and BKA. In vivo: 1% anthocyanin-rich blackcurrant extract in the diet for 12 weeks	Effects on osteoclast formation (TRAP assay, pit formation assay), osteoblast differentiation (alkaline phosphatase assay), bone histomorphometry analysis	Blackcurrant supplementation attenuated ovariectomy-induced bone loss in mice. BMD was higher in the OVX + BC group compared to OVX. Histological analysis showed improved trabecular bone parameters in the OVX + BC group. Osteoclast activity and differentiation were reduced by blackcurrant supplementation
Nagaoka et al. 2019 [31]	Suppression of osteoclast differentiation	petunidin	RAW264.7 cell line	>5 μg/ml	Significantly suppressed OCs’ differentiation, down-regulated expression of genes for c-Fos, NFATc1, matrix metalloproteinase 9 and cathepsin K	Petunidin significantly suppressed OCs’ differentiation

**Table 4:** Comparative summary of evidence direction across models.

Outcome	Human Studies	Animal Studies	In Vitro Studies	Overall Direction/Certainty
Bone Mineral Density (BMD)	↑ (modest, low–moderate certainty)	↑↑ (consistent, moderate strength)	—	Positive, low–moderate certainty
Bone Biomarkers (OCN, ALP, P1NP)	± (mixed)	↑ (consistent increases)	↑ (cell-level support)	Partially positive, low certainty
Osteoclast Activity	↓ (limited)	↓↓ (consistent inhibition)	↓↓↓ (strong inhibition)	Strong inhibition of resorption
Osteoblast Differentiation	↑ (suggestive)	↑↑ (enhanced in OVX models)	↑↑↑ (strong activation)	Strong stimulation of formation
Fracture Outcomes	Not assessed	↓ (indirect improvement)	—	Unclear, very low certainty

**Abbreviations:** BMD, Bone Mineral Density; ALP, Alkaline Phosphatase; OCN, Osteocalcin; OVX, Ovariectomized.

**Table 5:** Summary of findings and certainty of evidence according to GRADE for human studies.

Outcome	Population and Setting	Studies (Design)	What Was Measured	What the Studies Showed	Certainty (GRADE)	Why the Certainty Is Not Higher
Bone mineral density (BMD) at LS/TH/FN/whole-body	Adults, mostly postmenopausal women	2 cohorts; 2 RCTs	DXA BMD (site-specific)	Cohorts: higher anthocyanin intake associated with greater BMD; RCTs: modest ↑(up) in whole-body BMD/↑(up) net Ca retention	Low–Moderate	Small RCTs, short duration; residual confounding in cohorts; inconsistency across sites
Bone turnover biomarkers (P1NP, OC, CTX, ALP)	Adults	2 RCTs + 1 pilot	Standard serum markers	Mixed findings (some ↑(up) formation markers; others unchanged)	Low	Imprecision (small n), inconsistency, heterogeneity of products/doses
Fracture outcomes	Adults	None	Incident fractures	Not assessed	Very Low	No direct evidence

## Data Availability

All extracted data are provided within the article; additional files are available on request.

## References

[1] Hamidi M., Boucher B.A., Cheung A.M., Beyene J., Shah P.S. (2011). Fruit and vegetable intake and bone health in women aged 45 years and over: A systematic review. Osteoporos. Int..

[2] Brondani J.E., Comim F.V., Flores L.M., Martini L.A., Premaor M.O. (2019). Fruit and vegetable intake and bones: A systematic review and meta-analysis. PLoS ONE.

[3] Salvio G., Ciarloni A., Gianfelice C., Lacchè F., Sabatelli S., Giacchetti G., Balercia G. (2023). The Effects of Polyphenols on Bone Metabolism in Postmenopausal Women: Systematic Review and Meta-Analysis of Randomized Control Trials. Antioxidants.

[4] Feng R.C., Dong Y.H., Hong X.L., Su Y., Wu X.V. (2023). Effects of anthocyanin-rich supplementation on cognition of the cognitively healthy middle-aged and older adults: A systematic review and meta-analysis of randomized controlled trials. Nutr. Rev..

[5] Inchingolo A.D., Inchingolo A.M., Malcangi G., Avantario P., Azzollini D., Buongiorno S., Viapiano F., Campanelli M., Ciocia A.M., De Leonardis N. (2022). Effects of Resveratrol, Curcumin and Quercetin Supplementation on Bone Metabolism-A Systematic Review. Nutrients.

[6] Lin F., Chen J., Chen M., Lin S., Dong S. (2023). Protective effect and possible mechanisms of resveratrol in animal models of osteoporosis: A preclinical systematic review and meta-analysis. Phytother. Res. PTR.

[7] Shuid A.N., Abdul Nasir N.A., Ab Azis N., Shuid A.N., Razali N., Ahmad Hairi H., Mohd Miswan M.F., Naina Mohamed I. (2025). A Systematic Review on the Molecular Mechanisms of Resveratrol in Protecting Against Osteoporosis. Int. J. Mol. Sci..

[8] Bellavia D., Caradonna F., Dimarco E., Costa V., Carina V., De Luca A., Raimondi L., Fini M., Gentile C., Giavaresi G. (2021). Non-flavonoid polyphenols in osteoporosis: Preclinical evidence. Trends Endocrinol. Metab. TEM.

[9] Zeraattalab-Motlagh S., Ghoreishy S.M., Arab A., Mahmoodi S., Hemmati A., Mohammadi H. (2023). Fruit and Vegetable Consumption and the Risk of Bone Fracture: A Grading of Recommendations, Assessment, Development, and Evaluations (GRADE)-Assessed Systematic Review and Dose-Response Meta-Analysis. JBMR Plus.

[10] Sterne J.A., Savović J., Page M.J., Elbers R.G., Blencowe N.S., Boutron I., Cates C.J., Cheng H.Y., Corbett M.S., Eldridge S.M. (2019). RoB 2: A revised tool for assessing risk of bias in randomised trials. BMJ.

[11] Sterne J.A.C., Hernán M.A., Reeves B.C., Savović J., Berkman N.D., Viswanathan M., Henry D., Altman D.G., Ansari M.T., Boutron I. (2016). ROBINS-I: A tool for assessing risk of bias in non-randomised studies of interventions. BMJ.

[12] Hooijmans C.R., Rovers M.M., De Vries R.B., Leenaars M., Ritskes-Hoitinga M., Langendam M.W. (2014). SYRCLE’s risk of bias tool for animal studies. BMC Med. Res. Methodol..

[13] Schneider K., Schwarz M., Burkholder I., Kopp-Schneider A., Edler L., Kinsner-Ovaskainen A., Hartung T., Hoffmann S. (2009). ToxRTool—A new tool to assess the reliability of toxicological data. ALTEX.

[14] Hardcastle A.C., Aucott L., Reid D.M., Macdonald H.M. (2011). Associations between dietary flavonoid intakes and bone health in a Scottish population. J. Bone Miner. Res..

[15] Welch A., MacGregor A., Jennings A., Fairweather-Tait S., Spector T., Cassidy A. (2012). Habitual flavonoid intakes are positively associated with bone mineral density in women. J. Bone Miner. Res..

[16] Nosal B.M., Sakaki J.R., Macdonald Z., Mahoney K., Kim K., Madore M., Thornton S., Tran T.D.B., Weinstock G., Lee E.C.-H. (2022). Blackcurrants reduce the risk of postmenopausal osteoporosis: A pilot double-blind, randomized, placebo-controlled clinical trial. Nutrients.

[17] Hatcher K. (2017). The Effect of Whole Red Raspberry Juice on Bone Density and Biomarkers of Bone in Postmenopausal Osteopenic Women. Doctoral Dissertation.

[18] Hodges J.K., Maiz M., Cao S., Lachcik P.J., Peacock M., McCabe G.P., McCabe L.D., Cladis D.P., Jackson G.S., Ferruzzi M.G. (2023). Moderate consumption of freeze-dried blueberry powder increased net bone calcium retention compared with no treatment in healthy postmenopausal women: A randomized crossover trial. Am. J. Clin. Nutr..

[19] Li T., Wu S.M., Xu Z.Y., Ou-Yang S. (2014). Rabbiteye blueberry prevents osteoporosis in ovariectomized rats. J. Orthop. Surg. Res..

[20] Zheng X., Mun S., Gil Lee S., Vance T.M., Hubert P., Koo S.I., Lee S.-K., Chun O.K. (2016). Anthocyanin-rich blackcurrant extract attenuates ovariectomy-induced bone loss in mice. J. Med. Food.

[21] Melough M.M., Sun X., Chun O.K. (2017). The role of AOPP in age-related bone loss and the potential benefits of berry anthocyanins. Nutrients.

[22] Sakaki J., Melough M., Lee S.G., Kalinowski J., Koo S.I., Lee S.K., Chun O.K. (2018). Blackcurrant supplementation improves trabecular bone mass in young but not aged mice. Nutrients.

[23] Kaume L., Gilbert W.C., Brownmiller C., Howard L.R., Devareddy L. (2012). Cyanidin 3-O-β-D-glucoside-rich blackberries modulate hepatic gene expression, and anti-obesity effects in ovariectomized rats. J. Funct. Foods.

[24] Hong S., Kwon J., Song S., Park I., Jung D.S., Saruul E., Nho C.W., Kwon H.C., Yoo G. (2024). Suppressive Effects of Geoje Raspberry (Rubus tozawae Nakai ex JY Yang) on Post-Menopausal Osteoporosis via Its Osteogenic Activity on Osteoblast Differentiation. Nutrients.

[25] Devareddy L., Hooshmand S., Collins J.K., Lucas E.A., Chai S.C., Arjmandi B.H. (2008). Blueberry prevents bone loss in ovariectomized rat model of postmenopausal osteoporosis. J. Nutr. Biochem..

[26] Chen J.R., Lazarenko O.P., Wu X., Kang J., Blackburn M.L., Shankar K., Badger T.M., Ronis M.J. (2010). Dietary-induced serum phenolic acids promote bone growth via p38 MAPK/beta-catenin canonical WNT signaling. J. Bone Miner. Res..

[27] Zhang J., Lazarenko O.P., Blackburn M.L., Shankar K., Badger T.M., Ronis M.J., Chen J.R. (2011). Feeding blueberry diets in early life prevent senescence of osteoblasts and bone loss in ovariectomized adult female rats. PLoS ONE.

[28] Tanabe S., Santos J., La V.D., Howell A.B., Grenier D. (2011). A-type cranberry proanthocyanidins inhibit the RANKL-dependent differentiation and function of human osteoclasts. Molecules.

[29] Bickford P.C., Tan J., Shytle R.D., Sanberg C.D., El-Badri N., Sanberg P.R. (2006). Nutraceuticals synergistically promote proliferation of human stem cells. Stem Cells Dev..

[30] Bauer Y.G., Magini E.B., Farias I.V., Neto J.D.P., Fongaro G., Reginatto F.H., Silva I.T., Cruz A.C.C. (2024). Potential of Cranberry to Stimulate Osteogenesis: An In Vitro Study. Coatings.

[31] Nagaoka M., Maeda T., Moriwaki S., Nomura A., Kato Y., Niida S., Kruger M.C., Suzuki K. (2019). Petunidin, a B-ring 5′-O-Methylated Derivative of Delphinidin, Stimulates Osteoblastogenesis and Reduces sRANKL-Induced Bone Loss. Int. J. Mol. Sci..

